# Transcription Factors in Type 2 Diabetes: Molecular Mechanisms of Insulin Resistance and β-Cell Dysfunction

**DOI:** 10.2174/0115733998443779260129064444

**Published:** 2026-03-06

**Authors:** Seyyed Amin Seyyed Rezaei, Akbar Amirfiroozi, Moein Kohkalani, Maghsoud Mehri

**Affiliations:** 1 Department of Medical Genetics, Faculty of Medicine, Tabriz University of Medical Sciences, Tabriz, Iran

**Keywords:** Type 2 diabetes mellitus, T2DM, transcription factors, TFs, genetics, FOXO1, NF-κB

## Abstract

Type 2 diabetes mellitus (T2DM) is a common global disease affecting more than 588 million people. This number is projected to reach 852 million by 2050, imposing a significant socioeconomic burden. Type 2 diabetes results from insulin resistance (IR) in peripheral tissues and dysfunction of pancreatic beta cells. These two mechanisms and their proper functioning in the body are influenced by genetic and environmental factors. Transcription factors (TFs) play pivotal roles in these processes by regulating the expression of genes governing glucose and lipid metabolism. This study reviews the current knowledge on key transcription factors involved in the pathophysiology of type 2 diabetes. In the liver, FOXO1 enhances gluconeogenesis through upregulation of PEPCK and G6Pase expression under IR conditions, whereas SREBP1c and ChREBP enhance de novo lipogenesis and exacerbate hepatic steatosis and systemic IR. In adipose tissue, PPARγ coordinates adipocyte differentiation and insulin sensitivity, but phosphorylation at Ser273 impairs adiponectin expression. NF-κB stimulates chronic inflammation and links metabolic stress to insulin resistance. In skeletal muscle, MEF2 isoforms coordinate mitochondrial biogenesis and oxidative metabolism with PGC-1α, which are impaired in T2DM. Disruption of these transcription factors results in metabolic dysfunction. This highlights their potential as therapeutic targets. Therapeutic options such as FOXO1 inhibitors or PPARγ agonists are promising, although challenges such as off-target effects remain. Understanding the mechanisms by which transcription factors contribute to the pathophysiology of T2DM provides insights into novel strategies to reduce the progression and complications of T2DM.

## 1 INTRODUCTION

Type 2 diabetes mellitus (T2DM) is a chronic metabolic disorder characterized by persistent hyperglycemia. According to the International Diabetes Federation (IDF), 588.7 million people worldwide have diabetes, and this number is expected to increase to 852.5 million by 2050 [1]. The high prevalence of diabetes worldwide has imposed a significant economic, social, and psychological burden on patients, their families, society, and governments [2]. Given that type 2 diabetes accounts for approximately 90-95% of all diabetes cases, it is essential to elucidate the underlying pathophysiological mechanisms and develop robust strategies aimed at curbing its prevalence [3].

The development and progression of T2DM is primarily driven by two interrelated pathophysiological defects. These defects include insulin resistance (IR) and pancreatic beta-cell dysfunction [4]. Insulin resistance refers to the reduced responsiveness of key metabolic tissues, particularly skeletal muscle, liver, and adipose tissue, to insulin, resulting in impaired glucose uptake and utilization and failure to suppress hepatic glucose production [5]. Concurrently, beta-cell dysfunction results in the inability of these cells to secrete sufficient insulin to compensate for insulin resistance. This defect includes a relative deficiency of insulin secretion in response to elevated glucose levels and, over time, a progressive decline in beta-cell mass and function [6, 7]. Importantly, while insulin resistance creates a need for increased insulin production, beta-cell dysfunction results in this demand not being met, creating a cycle that ultimately leads to the persistent hyperglycemia that defines T2DM [4]. These two processes operate simultaneously and are among the fundamental drivers of disease progression.

A combination of environmental and genetic factors contributes to beta-cell dysfunction, IR, and ultimately T2DM. Environmental factors, such as excessive caloric intake, sedentary lifestyle, and obesity, exacerbate insulin resistance and beta-cell dysfunction, resulting in hyperglycemia [8]. Genetic predisposition also plays a role in risk. Estimates of heritability range from 40% to 70%, and numerous genome-wide association studies have identified important loci that influence insulin secretion and action, and energy homeostasis [9]. Understanding these genetic factors is critical for risk assessment, elucidation of the molecular mechanisms of beta-cell failure, and development of preventive and therapeutic interventions.

Transcription factors (TFs) are one of the crucial components of genetic factors, whose disruption can cause numerous problems. They are sequence-specific DNA-binding proteins that regulate gene expression by interacting with promoter and enhancer regions to either activate or repress transcription. TFs influence the assembly of the basal transcriptional machinery, determining when, where, and how much a gene is expressed. Structurally, TFs often contain distinct domains for DNA binding (such as homeodomains, zinc fingers, or basic helix-loop-helix (bHLH) motifs). They also have separate activation or repression domains that interface with co-regulators, chromatin remodelers, or other components of the transcriptional apparatus [10]. Because TFs occupy master-regulatory positions within gene networks, their dysregulation can give rise to diverse pathologies. In oncology, for example, mutations in TFs such as TP53 or MYC disrupt cell-cycle control and genomic stability, promoting tumorigenesis [11]. In neurodevelopmental disorders, alterations in TFs like MECP2 or SOX10 impair neuronal differentiation and synaptic function [12, 13]. Likewise, in T2DM, alterations in TFs that govern pancreatic β-cell identity (*e.g.*, regulators of insulin biosynthesis) or those modulating insulin sensitivity in peripheral tissues can precipitate β-cell failure and IR [14, 15]. Consequently, understanding the normal and pathological roles of TFs is essential for elucidating molecular mechanisms underlying T2DM and for identifying potential therapeutic targets.

This review aims to synthesize current knowledge on the roles of key transcription factors in driving the core pathophysiological defects of T2DM, IR in peripheral tissues and pancreatic β-cells dysfunction.

### 1.1 Search Strategy

A comprehensive literature search was performed using the PubMed, Scopus, and Web of Science databases. The search aimed to identify relevant studies on the role of transcription factors in the pathophysiology of type 2 diabetes mellitus (T2DM). The search strategy combined keywords and Medical Subject Headings (MeSH) terms, including: "Transcription Factors," "Insulin Resistance," "beta-cell dysfunction," "Pancreatic beta-cell," "Type 2 Diabetes Mellitus," "PPARgamma," "PDX1," "FOXO1," "SREBP1c," and "NF-kB." The search was limited to articles published in English. No date restrictions were applied to ensure a comprehensive historical perspective, but priority was given to seminal and recent high-impact studies for the final review.

## 2 TRANSCRIPTION FACTORS DRIVING INSULIN RESISTANCE

### 2.1 Liver-Centered TFs

#### 2.1.1 Forkhead Box O1 (FOXO1)

Forkhead box O1 (FOXO1) plays a central role in the regulation of hepatic glucose metabolism. It also functions as a promoter of gluconeogenesis and as a repressor of glycolysis. In the fasted state, deacetylated and/or unphosphorylated FOXO1 translocates to the nucleus, where it binds to insulin response elements in the PEPCK and G6Pase promoters. This binding directs their transcription and maintains endogenous glucose production at appropriate levels [16, 17]. After a meal, insulin activates the insulin receptor-IRS1-PI3K signaling cascade, leading to Akt activation. Activated Akt phosphorylates FOXO1 at several conserved residues, primarily Ser256, which are critical for 14-3-3 binding and nuclear export. Other sites include Thr24 and Ser319. Phosphorylation enhances the interaction of FOXO1 with 14-3-3 adaptor proteins, sequestering FOXO1 in the cytoplasm and preventing its translocation to the nucleus. Cytoplasmic retention targets FOXO1 for ubiquitin-mediated proteasomal degradation, thereby suppressing gluconeogenesis and enabling a metabolic switch to glycolysis (Fig. **1**). This regulatory mechanism ensures efficient glucose utilization and maintains systemic glucose homeostasis [18, 19].

In insulin-resistant and T2DM livers, multiple defects converge to impair Akt activation, serine phosphorylation of IRS1 by inflammatory kinases (*e.g.*, JNK), lipid-induced PKCθ activity, and endoplasmic reticulum stress, resulting in inadequate FOXO1 phosphorylation and its persistent nuclear retention. As a consequence, PEPCK and G6Pase remain aberrantly expressed, contributing to pathologic fasting hyperglycemia [20-23]. Counterbalancing Akt, serine/threonine phosphatases such as protein phosphatase 2A (PP2A) and protein phosphatase 1 (PP1) dephosphorylate FOXO1, restoring its DNA-binding capacity. Studies have shown that hepatic PP2A activity is elevated in diabetic rodent models and human patients, further prolonging FOXO1-driven gluconeogenesis [24, 25]. Together, the dysregulated interplay between kinases and phosphatases locks hepatocytes into a gluconeogenic phenotype despite nutrient abundance, highlighting FOXO1 as a nexus of hepatic insulin resistance and a promising target for therapeutic intervention.

#### 2.1.2 Sterol Regulatory Element-Binding Protein 1c (SREBP1c)

SREBP1c is the predominant isoform of SREBP1 in the liver and serves as a master transcriptional regulator of hepatic de novo lipogenesis (DNL) [26]. SREBP-1c, upon proteolytic activation and translocation to the nucleus, promotes transcription of genes encoding enzymes critical for fatty acid biosynthesis, such as Acetyl-CoA Carboxylase (ACC) (catalyzing the conversion of acetyl-CoA to malonyl-CoA), Fatty Acid Synthase (FAS) (mediating palmitate synthesis), and Stearoyl-CoA Desaturase 1 (SCD1) (introducing double bonds to form monounsaturated fatty acids) [27]. This coordinated transcriptional program promotes the conversion of dietary carbohydrates into triglycerides for energy storage, an adaptive response to postprandial nutrient excess under physiological conditions [28].

Early T2DM features a pathophysiological divergence in insulin pathway sensitivity wherein systemic IR in peripheral tissues (*e.g.*, skeletal muscle and adipose tissue) coincides with selective hepatic hypersensitivity. Compensatory hyperinsulinemia develops to counteract impaired glucose uptake in muscle and unrestrained lipolysis in adipose tissue [29]. Within the liver, however, insulin signaling exhibits tissue-specific discordance: while suppression of gluconeogenesis *via* the AKT-FOXO1 axis is blunted, contributing to fasting hyperglycemia, the mTORC1-SREBP1c lipogenic pathway retains heightened sensitivity to insulin stimulation [30]. This selective signaling enables hyperinsulinemia to potently activate SREBP1c through enhanced proteolytic processing and nuclear translocation, driving unrestrained DNL and triglyceride synthesis. Consequently, lipid accumulation generates lipotoxic metabolites (*e.g.*, diacylglycerols activating PKCε, ceramides inhibiting AKT) that further impair insulin receptor substrate (IRS) phosphorylation, establishing a self-reinforcing cycle that exacerbates hepatic steatosis, intensifies systemic IR, and accelerates metabolic dysfunction [31].

Direct SREBP1c inhibitors, such as photostatin, reduce hepatic lipogenesis and improve insulin sensitivity in rodent models [32, 33]. However, the clinical application of these inhibitors still has many challenges and limitations. The most important of these limitations is the concern about off-target effects on SREBP2 (regulating cholesterol biosynthesis). Furthermore, given the critical role of SREBP1c, there are additional concerns about its suppression [34]. Alternative approaches focus on upstream regulators, such as mTORC1 inhibitors, which suppress SREBP1c maturation but may enhance gluconeogenesis [35]. Thus, while SREBP1c is a compelling and significant target for the treatment of T2DM due to its pivotal role in pathogenic lipogenesis, there are several limitations and concerns that need to be considered and resolved before clinical trials.

#### 2.1.3 Carbohydrate Response Element Binding Protein (ChREBP)

Carbohydrate response element-binding protein (ChREBP) is a glucose-sensitive transcription factor. This protein links carbohydrate flux to both glycolysis and lipogenesis in hepatocytes. Increased flux through the pentose phosphate pathway increases xylulose-5-phosphate levels under high glucose conditions. This activates protein phosphatase 2A (PP2A) to dephosphorylate ChREBP and enhance its nuclear translocation and DNA binding to carbohydrate response elements (ChoREs) in target gene promoters [36, 37]. Once in the nucleus, ChREBP affects the glycolytic gene L-pyruvate kinase (PKLR). It also upregulates key lipogenic enzymes, including acetyl-CoA carboxylase (ACACA) and fatty acid synthase (FASN). Ultimately, it causes a coordinated increase in glycolysis and de novo fatty acid synthesis [38, 39].

In type 2 diabetes, chronic hyperglycemia locks ChREBP in a dephosphorylated, nuclear-activated state and chronically increases the expression of genes such as ACACA and FASN. The resulting increase in de novo lipogenesis contributes to triglyceride accumulation, dyslipidemia, and exacerbation of insulin resistance [38, 40]. The ability of ChREBP to drive both glycolytic and lipogenic programs suggests its role as a master metabolic switch. However, this dual action also suggests its pathological impact in diabetes. At first glance, ChREBP-induced upregulation of glycolytic enzymes may seem beneficial because increased glycolysis could help clear excess glucose from the bloodstream in type 2 diabetes. However, this accelerated glycolytic flux also produces large amounts of triose phosphate, which is diverted to de novo lipogenesis. Specifically, excess pyruvate and glycerol-3-phosphate produced during ChREBP-mediated glycolysis serve as substrates for ACACA and FASN, significantly increasing triglyceride synthesis in hepatocytes [41]. The resulting hepatic steatosis not only increases very low-density lipoprotein (VLDL) secretion and worsens systemic dyslipidemia, but also activates lipid-sensitive kinases such as protein kinase C epsilon (PKCε), which phosphorylate the insulin receptor and downstream signaling mediators at inhibitory sites. This disrupts insulin receptor signaling in the liver and skeletal muscle, and instead of reducing hyperglycemia, promotes insulin resistance [42, 43]. Thus, although the promotion of glycolysis by ChREBP initially appears to clear glucose, its association with lipogenesis transforms a seemingly adaptive response into a driver of metabolic dysfunction in T2DM (Fig. **2**).

### 2.2 Adipose-Tissue TFs

#### 2.2.1 Peroxisome Proliferator-Activated Receptor Gamma (PPARγ)

Peroxisome Proliferator-Activated Receptor Gamma (PPARγ) is a ligand-activated nuclear hormone receptor belonging to the steroid receptor superfamily. It forms a heterodimer with the retinoid X receptor (RXR) and binds peroxisome proliferator response elements (PPREs) in the regulatory regions of target genes. Highly expressed in adipose tissue, PPARγ is a master regulator of adipocyte differentiation and lipid metabolism [44, 45]. Upon activation by endogenous ligands (*e.g.*, fatty acids, eicosanoids) or synthetic agonists, PPARγ recruits co-activators (such as PGC-1α and CBP/p300) and displaces corepressors (NCoR, SMRT), thereby initiating transcription of genes essential for adipocyte maturation. These include fatty acid transporters (CD36, FATP1), lipogenic enzymes (FASN, DGAT1), and adipokines like adiponectin [46-48].

In obesity and T2DM, chronic nutrient excess elevates cyclin-dependent kinase 5 (Cdk5) activity in adipocytes, leading to aberrant phosphorylation of PPARγ at Ser273. This post-translational modification selectively downregulates insulin-sensitizing genes, most notably adiponectin, without blocking adipocyte differentiation, thereby contributing directly to systemic insulin resistance [49]. Thiazolidinediones (TZDs), including rosiglitazone and pioglitazone, bind PPARγ’s ligand-binding domain to prevent Ser273 phosphorylation. TZD therapy restores adiponectin levels, promotes redistribution of fat toward subcutaneous depots, and improves glycemic control. However, long-term use is limited by side effects such as weight gain, fluid retention (worsening heart failure), increased fracture risk, and rare hepatotoxicity [50].

#### 2.2.2 Nuclear Factor Kappa-light-chain-enhancer of Activated B Cells (NF-κB)

Nuclear Factor kappa-light-chain-enhancer of activated B cells (NF-κB) is a family of inducible transcription factors that play a central role in regulating genes involved in immune responses, inflammation, cell growth, and survival [51]. Increasing evidence suggests that chronic, low-grade inflammation is a significant contributor to the development and complications of T2DM. NF-κB, by controlling the expression of pro-inflammatory cytokines such as tumor necrosis factor-alpha (TNF-α), interleukin-1 beta (IL-1β), and interleukin-6 (IL-6), serves as a key molecular link that connects metabolic dysregulation with inflammatory signaling [52]. In the context of T2DM, multiple metabolic stressors, such as hyperglycemia, elevated free fatty acids, and oxidative stress, are known to activate the canonical NF-κB pathway in tissues crucial for glucose metabolism. These tissues include adipose tissue, the liver, skeletal muscle, and pancreatic islets [53]. Once activated, NF-κB translocates to the nucleus. In the nucleus, it stimulates the transcription of genes encoding cytokines, chemokines, and adhesion molecules, leading to a persistent state of chronic, low-level inflammation in metabolic tissues. This inflammatory microenvironment perpetuates insulin resistance by interfering with insulin receptor signaling. For example, cytokines such as TNF-α induce serine phosphorylation of insulin receptor substrate-1 (IRS-1), reducing insulin signaling and glucose uptake in muscle and adipose tissues [52]. Moreover, the inflammatory actions of NF-κB in adipose tissue contribute to macrophage infiltration and polarization towards a pro-inflammatory M1 phenotype (Fig. **3**). These adipose tissue macrophages and other immune cells further amplify local and systemic inflammation through paracrine and endocrine actions of cytokines, thereby exacerbating insulin resistance [54].

Clinical and experimental data strongly indicate that the inhibition of NF-κB signaling confers metabolic benefits in models of insulin resistance and T2DM. Pharmacological inhibitors of IKKβ (the kinase complex that activates NF-κB) or genetic ablation of NF-κB components improve insulin sensitivity and preserve β-cell function [55, 56]. Conversely, hyperactivation of NF-κB exacerbates the features of metabolic syndrome. A study conducted by Bin Yi *et al.* has demonstrated increased NF-κB activity in peripheral blood mononuclear cells of patients with T2DM. This activity correlates with markers of inflammation and insulin resistance [57].

Collectively, these findings underscore the role of NF-κB as a pivotal mediator that connects metabolic disturbances and inflammation in T2DM pathogenesis. Targeting the NF-κB signaling pathway continues to be a promising area of research for therapeutic intervention in T2DM and its complications.

### 2.3 Muscle-Centered TFs

#### 2.3.1 Myocyte Enhancer Factor 2 (MEF2)

Myocyte enhancer factor 2 (MEF2) transcription factors, which include the isoforms MEF2A, MEF2B, MEF2C, and MEF2D, are master regulators of muscle-specific gene programs and broader metabolic homeostasis. In skeletal muscle, MEF2 proteins cooperate with coactivators like peroxisome proliferator-activated receptor γ coactivator 1α (PGC-1α) to drive critical processes such as mitochondrial biogenesis and oxidative metabolism. Significantly, these MEF2-dependent processes are impaired in T2DM [58, 59].

MEF2 is a pivotal transcriptional regulator for the SLC2A4 gene in skeletal muscle. It encodes the insulin-responsive glucose transporter GLUT4. MEF2 binds to a conserved enhancer element in the GLUT4 promoter and cooperates with other factors, such as the GLUT4 enhancer factor (GEF), to drive basal and inducible transcription of SLC2A4. Disruption of MEF2 binding significantly reduces GLUT4 mRNA and protein levels [60, 61]. This fact emphasizes the necessity of MEF2 for maintaining glucose transport capacity. Under physiological conditions, MEF2 activity is dynamically regulated by insulin and exercise through opposing post-translational modifications. In response to insulin, the Ca2+/calmodulin-dependent phosphatase, calcineurin, is activated and dephosphorylates MEF2, increasing its activating potential on GLUT4 and other metabolic genes [62, 63]. In type 2 diabetes, chronic hyperglycemia and lipid overload disrupt calcineurin signaling, leading to defective MEF2 activation. This defect results in reduced SLC2A4 transcription, reduced GLUT4 protein in the sarcolemma, and impaired insulin-stimulated glucose uptake [64]. Therefore, restoring MEF2 activity, by enhancing calcineurin-mediated dephosphorylation, is a promising strategy to restore GLUT4-dependent glucose homeostasis in type 2 diabetes.

#### 2.3.2 Nuclear Respiratory Factor 1 (NRF1)

Nuclear respiratory factor 1 (NRF1) is a major transcriptional regulator that is critical for mitochondrial biogenesis and cellular energy metabolism. It acts as a metabolic gatekeeper by binding to the promoters of nuclear-encoded mitochondrial genes, such as TFAM, COX subunits, and TFB2M. It thereby drives mitochondrial biogenesis and increases oxidative phosphorylation capacity [65]. In metabolic disorders, NRF1 functions as a master regulator of nutrient sensing and energy balance. In the liver, hepatocyte-specific deletion of Nfe2l1 (encoding NRF1) causes fat accumulation, steatosis, inflammation, and progression to nonalcoholic steatohepatitis [66, 67]. This fact suggests its essential role in maintaining hepatic metabolic homeostasis. Beyond the liver, systemic NRF1 deficiency reduces fatty acid oxidation and mitochondrial OXPHOS components, leading to aberrant fat deposition in skeletal muscle and adipose tissue [68].

In addition to its role in mitochondrial biogenesis, NRF-1 has been shown to regulate carbohydrate metabolism by controlling the transcription of genes involved in this process. Carbohydrates are broken down to glucose, which, through glycolysis, supplies ATP for cellular functions. Impaired carbohydrate metabolism can precipitate T2DM [69]. NRF-1 can affect the pathogenesis of type 2 diabetes through the pathways contributing to carbohydrate metabolism. One of these pathways is through GLUT-4. It has been shown that NRF-1 can regulate the transcription of the myocyte enhancer factor 2 (MEF-2) gene. MEF-2 is a transcription factor that binds to the GLUT-4 promoter and regulates its transcription. Therefore, NRF-1 indirectly affects the expression of GLUT-4 [70]. GLUT4 is an insulin-responsive glucose transporter that mediates glucose uptake into muscle and adipose cells. Therefore, NRF-1 dysfunction can increase the risk of T2DM by disrupting the expression of GLUT-4. Another way in which NRF-1 is involved in the pathogenesis of T2DM is through lipid metabolism. Studies indicate that NRF-1 controls the gene-level expression of carnitine palmitoyl transferase 1 (CPT-1) and acetyl-CoA carboxylase (ACC) [71, 72]. These two enzymes are among the main enzymes of lipid metabolism. It has been shown that impaired lipid metabolism can lead to type 2 diabetes [69]. Therefore, NRF-1 can affect lipid metabolism and, in turn, the risk of developing type 2 diabetes by regulating the expression of these two enzymes.

## 3 TRANSCRIPTION FACTORS GOVERNING β- CELL FUNCTION

### 3.1 Pancreatic and Duodenal Homeobox 1 (PDX1)

During the development of the pancreas, Pancreatic and Duodenal Homeobox 1 (PDX1) is the first transcription factor to be expressed. Humans or mice that lack this factor have pancreatic agenesis, which means they cannot generate duct, exocrine, or endocrine cells [73-75]. The pancreas derives from an epithelial sheet of cells termed the definitive endoderm [76]. This definitive endoderm shares a common progenitor with the mesoderm, referred to as mesendoderm. The specification of the endoderm is influenced by various factors, including Wnt and Nodal signaling, which promote endoderm formation at elevated levels [77, 78]. The definitive endoderm subsequently folds into a rudimentary gut tube, which subsequently evolves into foregut endoderm [79]. Fibroblast growth factor (FGF), retinoic acid (RA), and the Wnt and Sonic hedgehog (Shh) signaling pathways have a role in the creation of foregut endoderm [80]. At that phase, PDX1 expression is observed, establishing it as one of the earliest transcription factors expressed in the developing pancreas. The initial expression of PDX1 coincides with the expression of SOX9, GATA4, FOXA2, and SOX17; however, around 30-33 days post-conception, SOX17 expression diminishes and is supplanted by NK Homeobox 6.1 (NKX6.1). This expression pattern of PDX1, SOX9, NKX6.1, and FOXA2 defines the multipotent pancreatic progenitor population, which gives rise to endocrine, exocrine, and ductal lineages [81].

PDX1 and ISL1 synergistically regulate the insulin promoter alongside the BETA2 protein in B cells. The link between PDX1-BETA2 and ISL1-BETA2 is, nevertheless, ambiguous. To examine the differential regulation of insulin gene expression by PDX1 and ISL1 under high versus normal glucose circumstances, co-immunoprecipitation (Co-IP) was conducted to determine whether PDX1-BETA2 or ISL1-BETA2 form distinct complexes in response to varying glucose signals. PDX1 interacted with BETA2 in HIT-T15 cells cultivated in high-glucose medium; however, no PDX1-BETA2 complex was formed in normal glucose medium. In contrast, the ISL1-BETA2 complex was detected solely in cells cultivated in standard glucose media. Collectively, PDX1, instead of ISL1, interacts with BETA2 to establish a PDX1-BETA2 complex in response to elevated glucose signals. Under normal glucose circumstances, the ISL1-BETA2 complex is established to sustain the basal level of insulin gene expression [82]. Therefore, PDX1, as a crucial component of the PDX1-BETA2 complex, plays a key role in controlling the elevated glucose levels and preventing T2DM. Furthermore, Diabetes is characterized by diminished expression of Slc2a2/Glut2 and compromised glucose-stimulated insulin secretion (GSIS) [83]. Consequently, the intake of glucose *via* Glut2 is a crucial factor in regulating GSIS in the diabetic condition. The transcription of Glut2 is regulated by Pdx1 [84]. The ectopic expression of Pdx1 alone induces Glut2, while the dominant negative inhibition of Pdx1 function or β-cell-specific genetic ablation of Pdx1 in mice significantly and selectively diminishes Glut2 expression, indicating that Pdx1 serves a crucial role in Glut2 regulation [83].

A key mechanism underlying beta-cell failure in T2DM is the suppression of Pdx1 by oxidative stress [85]. Oxidative stress compromises the survival and functionality of pancreatic β-cells, which is often seen as linked to the onset of diabetes mellitus and its consequences. The subsequent rise in oxidized biological components directly results in diseases such as impaired insulin secretion and the initiation of new cellular responses, specifically programmed cell death, or apoptosis [86]. It has been shown that in the islets of T2DM patients, cytoplasmic transposition and inactivation of transcription factors V-Maf Avian Musculoaponeurotic Fibrosarcoma Oncogene Homolog A (MAFA), NKX6.1, and PDX1 are induced by oxidative stress-related dysfunction of β-cells [87, 88]. Taking these together, the key role of this transcription factor in beta cell function and the pathogenesis of T2DM is clear.

### 3.2 MAFA

MAFA, also referred to as RIPE3b1, is an important member of the MAF family of basic leucine zipper proteins that plays a crucial role in regulating the expression of the insulin gene in pancreatic beta cells. It is recognized as a transcription factor that selectively binds to the conserved insulin enhancer region RIPE3b/C1-A2 and stimulates insulin gene expression [89]. MafA, together with PDX-1 and BETA2, acts synergistically to activate transcription of the insulin gene. Inhibiting the binding of PDX-1, BETA2, or MafA significantly diminishes insulin enhancer-driven expression in β cells, indicating that these factors are essential for the function of the β cells [90].

Pancreatic islets in T2DM show reduced GSIS capabilities compared to normal islets. This is accompanied by reduced mRNA and protein expression of MAFA [87, 91]. In therapeutic strategies, activation of MAFA expression is crucial for the generation of mature and functional beta cells from pluripotent stem cells. Furthermore, physiological activation of MAFA in human embryonic stem cells (hESCs) has been shown to increase GSIS [92]. MAFA also regulates a set of beta-cell-specific genes. It directly activates the expression of glucose transporter 2 (GLUT2/Slc2a2) and increases the expression of granulophilin (Sytl4) in pancreatic islets, as shown by the reduction of these genes in MafA knockout models [93, 94]. In Mafa knockout mice, pancreatic islets are intact at birth. However, beta cells over time show a deficiency in glucose-stimulated insulin secretion and undergo progressive degeneration by 8-12 weeks of age [95, 96]. These data suggest that MAFA controls beta cell development, maintenance of function, and survival.

### 3.3 NKX6.1

NKX6.1, a homeobox-containing transcription factor, was originally discovered and isolated from the chromosome 4 of islet of Langerhans and insulinoma cell lines [97]. NKX6.1 is expressed in early stages of pancreatic development as well as in mature beta cells. This transcription factor plays several critical roles in pancreatic differentiation and function [98]. In the developing pancreas, there are three distinct NKX6.1+ cell types: pancreatic progenitor cells (PDX1+/NKX6.1+), endocrine progenitor cells (NGN3+/NKX6.1+), and mature pancreatic beta cells (insulin+/PDX1+/NKX6.1+). This presence in a variety of cell types during pancreatic development suggests a critical role for NKX6.1 in pancreatic cells [99].

NKX6.1 is primarily expressed in beta cells. It is critical for their formation and functional maturation. The gene is essential for the development of insulin-producing beta cells as well as for the maintenance of their glucose-stimulated insulin secretion [100]. Previous studies in rodents have shown that NKX6.1 expression is critical for the maintenance of pancreatic beta cell identity *in vivo* and that its overexpression leads to increased GSIS [101]. A separate study showed that NKX6.1 directly regulates genes involved in insulin processing, including Ero1lb and Slc30a8. In addition, essential genes governing beta cell function, glucose uptake, and metabolic processes are regulated by NKX6.1 expression [102]. Furthermore, NKX6.1 is critical for the maintenance of beta cell identity. Studies have shown that suppression of NKX6.1 in adult mouse beta cells results in the expression of NGN3. NGN3 expression converts these cells into Sst-expressing delta cells, thus indicating a change in beta cell identity due to the lack of NKX6.1 [101]. These findings are consistent with results obtained in T2D models, in which beta cells transform into alternative islet cells [103]. These findings indicate that NKX6.1 is a critical transcriptional regulator that coordinates some essential functions of beta cells.

### 3.4 Signal Transducer and Activator of Transcription 3 (STAT3)

STAT3, a central member of the JAK-STAT transcription factor family, plays complementary roles in preserving pancreatic β-cell function. Studies on preclinical models (cells and mice) have shown that their dysregulation contributes to the β-cell failure that underlies T2DM. STAT3 has been shown to regulate mitochondrial gene expression and mitochondrial function in β-cells, linking cytokine and growth-factor signaling to the bioenergetic and secretory competence required for glucose-stimulated insulin secretion [104]. Recent mechanistic work by Xiaoting Hou *et al.* further emphasizes the role of STAT3 at β-cell mass control [105]. Talin-1 prevents Smurf1-mediated ubiquitination and proteasomal degradation of STAT3, sustaining STAT3 levels that are necessary for β-cell proliferation and maintenance of insulin expression. Loss of this axis impairs glucose tolerance in mice. Moreover, recent preclinical studies indicated that IL-22 signals *via* IL-22RA1 to activate STAT3 in pancreatic β cells, promoting mitochondrial fitness and preserving β-cell identity and insulin secretion [106, 107]. However, these effects are shown in mouse models and ex vivo human islets and are still awaiting robust *in vivo* validation in humans.

### 3.5 c-Myc

c-Myc is a potent growth-promoting TF in β-cells, but its effects depend on the level of expression. Myc is a pleiotropic transcription factor that regulates a wide range of cellular processes, like proliferation, growth, death, differentiation, and the stability of the genome [108, 109]. In cells that are in a quiescent state, Myc is expressed at extremely low levels, if at all. During normal development, growth, and physiology, these normally low levels experience slight increases that range from 1.5 to 2 times their normal value [110]. Elevated Myc levels in β-cells result in enhanced β-cell proliferation and death, decreased insulin gene expression, and the onset of diabetes [111]. Conversely, the mild increase of Myc expression in rodent and human β-cells promotes β-cell replication without triggering cell death or diminishing insulin production, indicating that optimal levels of Myc may possess therapeutic potential for β-cell regeneration [112]. Harmine, a moderate inducer of Myc expression, significantly promotes human β-cell proliferation both *in vitro* and *in vivo*, without evidence of β-cell death or dedifferentiation [113]. Thus, while an excessive amount of Myc can be damaging to the β-cell, it is essential to have modest physiological levels of Myc in order to ensure that the β-cell still functions normally [114].

## 4 MECHANISMS OF TF DYSREGULATION IN β- CELLS

### 4.1 Glucotoxicity & Lipotoxicity

Chronic exposure to elevated glucose and free fatty acids imposes sustained endoplasmic reticulum (ER) and oxidative stress on pancreatic β-cells, provoking activation of canonical unfolded protein response (UPR) branches (PERK-eIF2α-ATF4-CHOP, IRE1-XBP1, and ATF6) and stress kinases such as c-Jun N-terminal kinase (JNK). These adaptive pathways, when persistently engaged by metabolic overload, transition to maladaptive signaling that impairs the transcriptional networks required for insulin biosynthesis, secretion, and β-cell identity [115, 116]. Activation of ATF6 during prolonged ER stress has been linked to repression of β-cell-enriched transcription factors and the insulin gene. Mechanistic work indicates that ATF6 can upregulate the orphan nuclear receptor small heterodimer partner (SHP/NR0B2), which in turn represses key TFs of the β-cell program, including PDX1, NEUROD1, and MAFA, thereby reducing insulin gene expression and secretory competence. This ATF6→SHP axis provides a direct molecular route by which ER stress can downregulate critical lineage TFs [117, 118].

Oxidative stress and reactive oxygen species (ROS), common downstream mediators of glucolipotoxicity, promote activation of JNK. JNK signaling diminishes PDX1 DNA-binding activity and contributes to its cytoplasmic mislocalization [119]. Moreover, sustained ER stress elevates CHOP (DDIT3), which marks a shift from adaptive to apoptotic UPR signaling. CHOP induction is associated with reduced expression and activity of β-cell TFs and with mitochondrial dysfunction, secretory failure, and increased β-cell apoptosis; genetic or pharmacologic reduction of CHOP signaling preserves β-cell function in multiple experimental models. Thus, prolonged CHOP upregulation links maladaptive UPR to both transcriptional repression of β-cell identity genes and loss of viable β-cell mass [120, 121].

### 4.2 Epigenetic Alterations in β-Cells

Chronic metabolic stress induces stable and locus-specific epigenetic changes in pancreatic β-cells that contribute to reduced insulin expression and loss of β-cell identity. Studies of human islets and animal models have shown increased DNA methylation at regulatory regions of PDX1 and other β-cell genes in donors with type 2 diabetes, which correlates with decreased PDX1 mRNA and impaired insulin secretion. These genome-wide methylation shifts are enriched at enhancers and at sites bound by β-cell transcription factors, supporting a model in which altered cytosine methylation reduces accessibility of key regulatory elements and represses lineage-defining gene programs [122, 123].

Histone deacetylation is another epigenetic mechanism that plays a crucial role in T2DM. Among class I HDACs, HDAC3 is a viable target for β-cell function. A genetic deletion of HDAC3 [124] and the HDAC3 inhibitor BRD3308 [125] significantly improved β-cell function by increasing insulin secretion. Additionally, class II HDACs found in the cytoplasm and nucleus contribute to β-cell function. Genetic deletion of class II HDACs (HDAC4/5/9) increases β and δ cell numbers, while overexpression decreases them [126].

Long noncoding RNAs (lncRNAs) can influence the expression of protein-coding genes at both the transcriptional and post-transcriptional stages of the biochemical process [127]. MALAT1 is an lncRNA that has a role in the transcriptome of β cells. It was initially described as a nuclear lncRNA that regulates alternative splicing and is highly expressed [128]. In β-cells, MALAT1 stabilizes polypyrimidine tract binding protein 1 (PTBP1), which in turn enhances PDX1 mRNA stability and supports insulin secretion [129]. Collectively, these findings position MALAT1 as a key modulator of β-cell functional capacity, though its complete ablation results in only mild metabolic impairment.

### 4.3 Auto/Paracrine Cytokine Effects

The dysfunction of pancreatic β-cells induced by proinflammatory cytokines is associated with a reduction in the expression of β-cell-specific transcription factors and genes, including insulin [130]. The interleukins IL-1α and IL-1β are potent inflammatory cytokines that signal through the IL-1R1/IL-1RAcP receptor complex and amplify immune responses. Although macrophages are a major source, other immune and non-immune cell types can also produce IL-1 family cytokines [131]. Genetic deletion or pharmacological blockade of IL-1 signaling often enhances insulin secretion, whereas exposure of islets to IL-1α/IL-1β (particularly under stress conditions such as hypoxia or prolonged exposure) suppresses insulin gene expression and secretion. Activation of the NF-κB pathway downstream of IL-1R1 contributes to cytokine-mediated β-cell dysfunction by repressing insulin gene transcription, in part *via* down-regulation of the key insulin transcription factors PDX-1 and MafA [132-134]. TNF-α similarly activates NF-κB and can impair β-cell function [135]. In addition, IL-1β and IFN-γ reduce ER Ca^2+^ handling (*e.g.*, SERCA activity), promoting ER stress and impairing glucose-stimulated insulin secretion [136, 137]. IL-1β also activates stress kinases such as JNK, which contribute to β-cell dysfunction and apoptosis, and can promote nuclear localization of FOXO1, thereby diminishing PDX-1 expression [130, 138].

## 5 THERAPEUTIC IMPLICATIONS & TF-TARGETED STRATEGIES

### 5.1 PPARγ Agonists (TZDs) & Beyond

Pioglitazone and rosiglitazone, members of the thiazolidinedione (TZD) class, are potent PPARγ activators in adipose tissue that enhance insulin sensitivity by inducing PPARγ-regulated gene expression and promoting the redistribution of fat from visceral and ectopic depots into subcutaneous stores, which translates clinically into improved glucose tolerance but is consistently accompanied by predictable side effects such as weight gain and fluid retention due to increased subcutaneous fat expansion and renal sodium reabsorption [139]. To retain the metabolic benefits of PPARγ activation while minimizing adverse outcomes, selective PPARγ modulators (SPPARMs) have been developed as partial agonists or selective ligands that engage PPARγ differently, often using non-TZD scaffolds, to uncouple insulin sensitization from toxicity [140]. For example, INT131, a prototype SPPARM, lowered fasting glucose in a short Phase II study comparably to rosiglitazone but did not produce the weight gain or edema seen with TZDs [141]. However, the long-term efficacy/safety of these drugs remains to be established.

### 5.2 Modulators of the FOXO1/SIRT1 Axis

SIRT1 Activators (*e.g.*, Resveratrol, SRT2104/SRT1720) are deacetylases that modify FOXO1 and other metabolic TFs. In hepatocytes, SIRT1-mediated deacetylation of FOXO1 alters its activity on gluconeogenic genes. Activating SIRT1 (*e.g.*, with resveratrol or synthetic agonists like SRT2104) was hypothesized to reduce hepatic gluconeogenesis. Indeed, animal studies suggest SIRT1 activators improve metabolic profiles [142], but human trials have been disappointing. In a 28-day trial of SRT2104 in T2D patients, no consistent dose-related changes in fasting glucose or insulin were seen [143]. Similarly, meta-analyses of resveratrol trials report that high-dose resveratrol (≥500-1000 mg/day) did not significantly improve glucose or HbA1c in older T2D patients [144]. These mixed results likely reflect challenges of poor bioavailability, rapid metabolism, and off-target actions of SIRT1 activators.

On the other hand, Small-Molecule FOXO1 Inhibitors directly inhibit FOXO1 DNA-binding, offering another approach to blunt hepatic glucose output. Recent preclinical studies have identified novel FOXO1 inhibitors with high specificity. In diabetic mice, a lead compound (compound 10) selectively blocked FOXO1 and improved insulin sensitivity and glucose control without causing weight gain [145]. These inhibitors reduced the expression of key gluconeogenic enzymes in the liver and lowered fasting glucose in rodents. Early compounds like AS1842856 were found to have off-target effects, but newer molecules are more specific. Although human trials are not yet available, these data support FOXO1 blockade as a promising strategy to decrease hepatic gluconeogenesis.

### 5.3 HDAC Inhibitors

In animal models of diabetes, HDAC inhibitors can restore β-cell gene expression under stress. For instance, the pan-HDAC inhibitor ITF2357 (givinostat) protected mouse islets from inflammatory damage. Oral ITF2357 normalized hyperglycemia in STZ-treated mice, improved islet function, and enhanced glucose-stimulated insulin secretion [146]. This was linked to reduced inflammatory NO production in β-cells. However, broad HDAC inhibition can also be deleterious. Recent studies have found that pan-HDACi, such as trichostatin A (TSA), dramatically alter β-cell chromatin. They increased global histone acetylation, impairing β-cell identity [147]. In that report, TSA-treated β-cells showed reduced Pdx1/Mafa expression and defective insulin secretion. Thus, while certain HDACi can enhance β-cell survival under cytokine stress, indiscriminate HDAC inhibition risks off-target gene changes and cytotoxicity. To date, no HDAC inhibitor is approved for T2D (their current clinical use is primarily in oncology).

### 5.4 Emerging Gene-Editing & RNAi Approaches

Cutting-edge gene-editing tools offer the possibility of tissue-specific TF modulation. In concept, CRISPR activation (CRISPRa) could be used to upregulate beneficial TFs (*e.g.*, PDX1) or CRISPR interference (CRISPRi) to silence deleterious ones (*e.g.*, FOXO1) in a cell-specific manner. Proof-of-concept studies (mostly in cultured cells and rodent models) have demonstrated that delivering dCas9-based activators or repressors can markedly alter target gene expression. For example, delivery of Pdx1-activating CRISPRa to β-cell precursors enhances insulin gene transcription [148]. However, these approaches remain experimental. Major hurdles include safe and specific delivery. Viral vectors (like AAV) can target the pancreas or liver but may cause immune responses or insertional mutagenesis, whereas newer nonviral carriers (lipid nanoparticles) have yet to achieve sufficient cell-type specificity. No human trials of CRISPRa/i for diabetes are underway, but animal studies are laying the groundwork.

Antisense strategies to knock down TFs have shown metabolic benefits in rodents. A classic example is liver-directed inhibition of Foxo1. In obese insulin-resistant mice, Foxo1 antisense oligonucleotides (ASO) therapy markedly suppressed hepatic gluconeogenic genes (PEPCK, G6Pase) and lowered fasting glucose by ~30-50% [149]. These mice also had reduced liver fat and improved insulin sensitivity. Such studies confirm that reducing FOXO1 in the liver can normalize glycemia. However, RNAi/ASO therapies can elicit innate immune activation (via toll-like receptors) and off-target hybridization if not carefully designed [150]. Ensuring delivery specifically to the liver (for gluconeogenesis control) remains an important area with potential for improvement. Chemical modifications and targeting ligands are helpful, but their clinical use in T2D has yet to be demonstrated.

## 6 FUTURE DIRECTIONS & KNOWLEDGE GAPS

In the long term, there may be a need to personalize TF-targeted therapies. A dominant TF signature could potentially stratify patients. For example, some individuals may have a metabolic disorder driven by hepatic ChREBP. In contrast, others may have diabetes driven by NF-κB-mediated inflammation. Understanding such subgroups could guide tailored interventions. In parallel, the study of TFs could lead to the identification of novel and effective biomarkers. Circulating TF fragments or molecules regulated by TFs could indicate impending β-cell failure. To date, several biomarkers are good predictors of type 2 diabetes, but TFs have not received sufficient attention in this context. Among the biomarkers identified are microRNAs. In youth-onset type 2 diabetes, basal levels of some circulating microRNAs (*e.g.*, miR-155, miR-130b, miR-126) have been shown to predict rapid decline in β-cell function [151]. Therefore, a deeper understanding of the role of transcription factors in type 2 diabetes may help patients with this disease in several ways.

Furthermore, the use of TFs as therapeutic targets poses significant safety challenges due to their broad gene effects. For example, PPARγ agonists (TZDs) improve insulin sensitivity but cause bone loss by increasing bone marrow adipogenesis [152].To reduce off-target effects, tissue-specific drug delivery systems are crucial. A recent study by Alexandre J Debacker *et al.* [153] showed that the attachment of nucleic acid drugs to N-acetylgalactosamine (GalNAc) directs them to hepatocytes *via* the ASGPR receptor, allowing for liver-specific inhibition. The development of similar targeted drug delivery platforms for TF modulators (small molecules, siRNAs, *etc.*) will be key to achieving therapeutic specificity and minimizing side effects.

## CONCLUSION

The review suggests that particular transcription factors have significant effects on T2DM. These transcription factors include those that regulate gene expression in the liver, such as FOXO1, SREBP1c, and ChREBP. The PPARγ regulates gene expression in adipose tissue. Those that regulate gene expression in β-cells include PDX1, Mafa, Nkx6.1, and STAT3. The effects of their potential deregulation include increased gluconeogenesis, lipogenesis, and inflammation, as well as β-cell dedifferentiation. We have also discussed other promising therapeutic strategies. These include PPARγ modulators, FOXO1 inhibitors, HDAC inhibitors, and RNA and gene editing. Although these strategies show potential, they are limited by problems such as off-target effects. To overcome these problems, these strategies need to be validated with human multi-omics data and tested for their safety.

## Figures and Tables

**Fig. (1):**
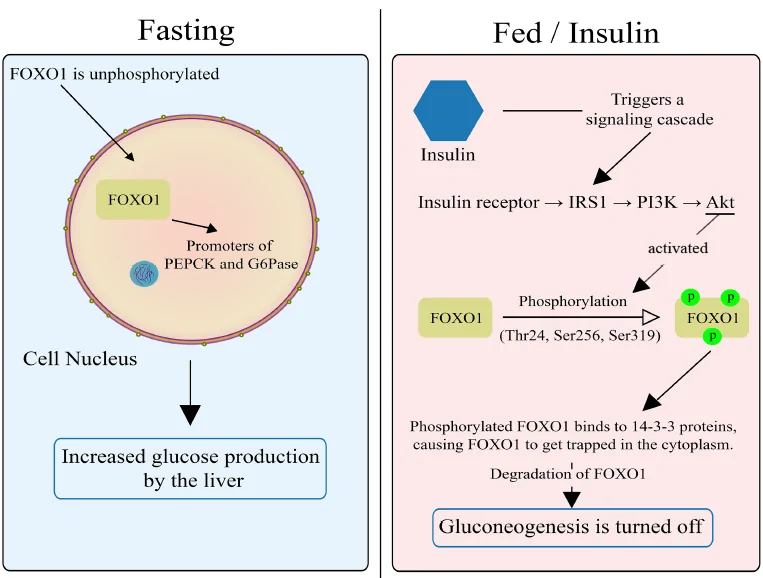
Central role of FOXO1 in hepatic glucose metabolism.

**Fig. (2):**
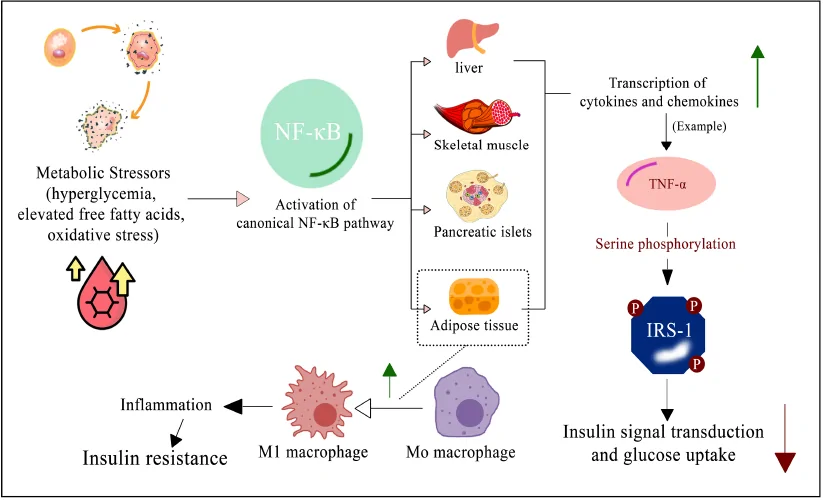
Role of ChREBP in Insulin Resistance.

**Fig. (3):**
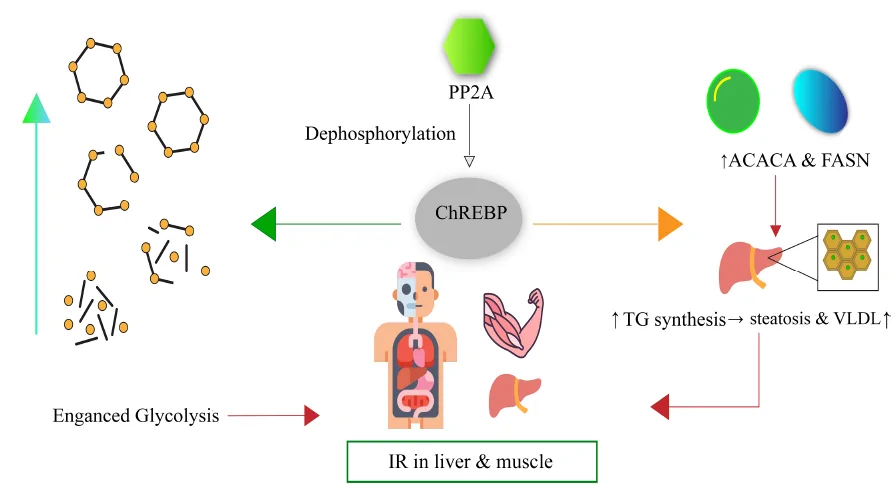
Role of NF-kΒ in Type 2 Diabetes and Insulin Resistance. IRS1: Insulin receptor substrate 1.

## LIST OF ABBREVIATIONS

ACACA: Acetyl-CoA Carboxylase
ChREBP: Carbohydrate Response Element Binding Protein
ER: Endoplasmic Reticulum
G6Pase: Glucose-6-phosphatase
HDAC: Histone Deacetylase
IFN-γ: Interferon-gamma
IL-1β: Interleukin-1 Beta
IR: Insulin Resistance
JAK-STAT: Janus Kinase-signal Transducer and Activator of Transcription Pathway
MAFA: V-maf Musculoaponeurotic Fibrosarcoma Oncogene Homolog A
MEF2: Myocyte Enhancer Factor 2
NF-κB: Nuclear Factor Kappa-light-chain-enhancer of Activated B Cells
NRF1: Nuclear Respiratory Factor 1
PDX1: Pancreatic and Duodenal Homeobox 1
PPARγ: Peroxisome Proliferator-activated Receptor Gamma
SREBP1c: Sterol Regulatory Element-binding Protein 1c
STAT3: Signal Transducer and Activator of Transcription 3
TF(s): Transcription Factor(s)
TNF-α: Tumour Necrosis Factor-alpha
T2DM: Type 2 Diabetes Mellitus
c-Myc: Cellular Myelocytomatosis Oncogene
